# Senator Hill

**Published:** 1882-04-20

**Authors:** 


SENATOR HILL, of Georgia, has had a third operation performed for the removal of a cancerous affection, by Prof. Gross, of Philadelphia. The last operation consisted in the removal of a portion of the salivary gland.

